# Postoperative effects of laparoscopic sleeve gastrectomy in morbid obese patients with type 2 diabetes

**DOI:** 10.1186/s40064-016-2167-8

**Published:** 2016-04-22

**Authors:** Mehmet Mihmanli, Riza Gurhan Isil, Emre Bozkurt, Uygar Demir, Cemal Kaya, Ozgur Bostanci, Canan Tulay Isil, Pinar Sayin, Sibel Oba, Feyza Yener Ozturk, Yuksel Altuntas

**Affiliations:** General Surgery Clinic, Sisli Hamidiye Etfal Education and Research Hospital, Halaskargazi Cad. Etfal Sok., 34371 Sisli, Istanbul Turkey; Anesthesiology and Reanimation Clinic, Sisli Hamidiye Etfal Education and Research Hospital, Halaskargazi Cad. Etfal Sok., 34371 Sisli, Istanbul Turkey; Endocrinology Clinic, Sisli Hamidiye Etfal Education and Research Hospital, Halaskargazi Cad. Etfal Sok., 34371 Sisli, Istanbul Turkey

**Keywords:** Morbid obesity, Laparoscopic sleeve gastrectomy, Type 2 diabetes, HbA1c, Diabetes remission criteria

## Abstract

**Background:**

Laparoscopic Sleeve Gastrectomy has become one of the most popular bariatric surgery types and helps treating not only obesity but also endocrinological diseases related to obesity. Therefore we aimed to evaluate the effects of laparoscopic sleeve gastrectomy on the treatment of type 2 diabetes.

**Methods:**

All patients, who underwent morbid obesity surgery during 2013–2014 and had a HbA1c >6 % were included in this prospective study. Demographical data, usage of oral antidiabetic drugs or insulin were recorded, and laboratory findings as HbA1c and fasting plasma glucose were evaluated preoperatively and postoperatively at the 6th and 12th months. Diabetes remission criteria were used to assess success of the surgical treatment.

**Results:**

Totally 88 patients were included in this study. 55 patients were using oral antidiabetic drugs and 33 patients were using insulin. At the 6th month complete remission was observed in 80 (90.9 %), partial remission in 3 (3.4 %) and persistent diabetes in 5 (5.6 %) patients. At the 12th month complete remission was observed in 84 (95.4 %), partial remission in 1 (1.1 %) and persistent diabetes in 3 (3.4 %) patients.

**Conclusions:**

This study indicated that laparoscopic sleeve gastrectomy surgery achieved a complete remission of diabetes in 95.4 % patients having type 2 diabetes during a 1 year fallow up period. However, complete remission of type 2 diabetes has been reported as 80 % during long term fallow up in the literature. In our opinion this rate may change with longer follow up periods and studies involving more patients suffering type 2 diabetes.

## Background

Every year 100,000 people die because of diabetes in the world (http://www.who.int/bulletin/volumes/86/10/07-048785/en/). Type 2 diabetes is commonly a component of metabolic disorders in the morbid obese patient (http://www.who.int/gho/ncd/risk_factors/obesity_text/en/). It is estimated, that 35.8 million (2.3 %) people in the world are diagnosed to have overweight or obesity (http://apps.who.int/gho/data/node.main.A865DIABETES?lang=en).

This serious health issue deserves to attract attention not only by internal specialists, but also by surgeons. Providentially recent studies reported surgical success up to 89 % in the treatment of type 2 diabetes in the morbid obese patients (Buchwald et al. 2004). Bariatric surgery may fill the gap between medical treatment and complete remission in type 2 diabetes. However, bariatric surgery varies from minimal surgical interventions as laparoscopic gastric plication and laparoscopic sleeve gastrectomy (LSG) to major surgical interventions as laparoscopic Roux-en-Y Gastric By-pass (RYGBP), laparoscopic mini gastric bypass and duodenal switch procedures. Especially, achieves good long-term results including weight-loss maintenance, reduction of comorbidities and improvement of quality of life (Cho et al. 2015).

Laparoscopic sleeve gastrectomy has gained popularity among surgeons, because it is a relative minimally invasive procedure, easy to do and requires a short procedure time compared to gastric bypass (Torgersen et al. 2014).

The purpose of this study was to evaluate postoperative effects of LSG in morbid obese patients with type 2 diabetes, to present our one-year follow-up and to underline possible advantages of LSG.

## Methods

This study was approved by a governmental ethical committee (Sisli Hamidiye Etfal Education and Training Hospital Ethical Committee Registration Number: 563). Verbal and written informed consent was obtained from all patients at the study beginning. Study design was a prospective clinical trial during the years 2013–2014 including all patients, who applied to our bariatric surgery polyclinic, because of morbid obesity and who had type 2 diabetes. Inclusion criteria were as follows: being morbid obese (BMI > 40 m^2^/kg), having type 2 diabetes and or having a glycosylated hemoglobin (HbA1c) >6 %, undergoing LSG. Excluded were patients who did not come to routine controls and who didn’t give informed consent for the study. Demographical data was recorded. HbA1c and fasting plasma glucose (FPG) were evaluated preoperatively and at the postoperatively 6th and 12th months. Patients were questioned, if they were taking insulin or oral antidiabetic drugs. Diabetes age was calculated. After bariatric surgery diabetes remission criteria of American Diabetes Association (ADA: complete remission is achieved when HbA1c is <6 % without any medication, partial remission is achieved when HbA1c is 6–6.5 % without any medication and not remission is a HbA1c > 6.5 % without any medication or using any medication for diabetes regulation) were used to assess the success of the treatment (Mingrone et al. 2012; Standards of Medical Care in Diabetes–2012 2012). Excess weight loss (EWL) was calculated for the 6th and 12th months.

### Surgical technique

All patients were given the “French” position before surgery, where the primary surgeon stood between the patient’s legs, first assistance stood at the primary surgeon’s right side and second assistance stood at his left side. Five ports were used: First a 10-mm trocar was placed 2 cm to the left of the midline above the umbilicus for optical view, then a 15-mm trocar was placed into the right midclaviculer-line, then a second 10-mm trocar was placed into the left midclavicular-line, afterwards a 5-mm trocar was placed into the sub-xiphoid area as liver retractor and at last a second 5-mm trocar was placed into the left subcostal area to pull the stomach.

The stomach was completely mobilized from the greater omentum side, beginning at the line of incisura angularis by LigaSure™ (Covidien, USA). At first proximal dissection was performed up to the angle of His, distal dissection was performed until to the pylorus. Then a 36 F bougie was inserted by the anesthesiology team along the lesser curvature of the stomach. Antral resection was started 2–4 cm from the pylorus and continued up to 0.5–1 cm medial to the angle of His. Hemostasis was checked and provided by Endoclips™ (Covidien, USA). Possible leakage was checked with methylene blue in saline given through the bougie.

### Statistical analysis

Statistical analyses were made with SPSS 22 (SPSS Inc. Chicago). Data was presented as mean ± standard deviation and categorical variables were expressed as frequencies.

## Results

Totally 89 patients with type 2 diabetes, who underwent LSG in our clinic during the years 2013–2014 were eligible for this study. One patient was excluded because she did not come to routine controls and 88 patients were included into the study. There were 28 (31.81 %) male and 60 (68.18 %) female patients with a mean age of 36 ± 9.35 (Table 1). The mean follow up of these 88 patients was 16.09 ± 3.32 months (minimum 12–maximum 24 months).

**Table 1:** Demographic data

	n = 88
Patient age (years) Mean ± SD	36 ± 9.3
Sex (female/male) n (%)	60/28 (68.19/31.81 %)
BMI (kg/m^2^) Mean ± SD	48.65 ± 7.71

*BMI* body mass index

Preoperative HbA1c of the 88 patients was 7.33 ± 1.24 %, at the 6th month HbA1c was 5.5 ± 0.7 % and at the 12th month HbA1c was 5.2 ± 033 %. Preoperative FBG of the 88 patients was 166.85 ± 50.38 mg/dL, at the 6th month FBG was 92.29 ± 23.28 mg/dL and at the 12th month FBG was 87.94 ± 7.02 mg/dL. Preoperative BMI of the 88 patients was 48.65 ± 7.71 kg/m^2^, BMI at the 6th month was 30 ± 3.28 kg/m^2^ and BMI at the 12th month was 26 ± 2.53 kg/m^2^ with a 6th month EWL of 68 ± 5.87 % and 12th month EWL of 81 ± 4.98 % (Table 2).

**Table 2:** Preoperative and postoperative patients’ data

	Preoperative	Postoperative 6th month	Postoperative 12th month
HBA1C (%) mean ± SD	7.33 ± 1.24	5.5 ± 0.7	5.2 ± 0.33
FPG (mg/dL) Mean ± SD	166.85 ± 50.38	92.29 ± 23.42	87.94 ± 7.02
EWL (%) mean ± SD		68 ± 5.87	81 ± 4.98

*FGL* fasting plasma glucose, *HbA1C* glycosylated hemoglobin, *EWL* estimated weight loss

Preoperative 33 patients were using insulin, 35 patients were using oral antidiabetic drugs, 20 patients did not take any medications and were diagnosed to have type 2 diabetes during preoperative assessment; endocrinology prescribed them to use oral antidiabetic drugs. Type 2 diabetes age of 33 patients who were using insulin were 39.42 ± 15.52 months, 35 patients who used oral antidiabetic medicine were 20.2 ± 9.26 months and 20 patients who were started to use oral antidiabetic drugs during preoperative assessment were 9.6 ± 3.34 months (diabetes age was calculated from the diagnose date to the operation date, it takes about 6–18 months to have an operation appointment in our clinic). At the 6th month of postoperative follow-up 34 of 35 patients, who used oral antidiabetic drugs were in complete remission, 26 of 33 patients who were using insulin were in complete remission. 2 of 33 patients were in partial remission and 5 of 33 patients were not in remission. 19 of 20 patients who were prescribed to use oral antidiabetic drugs during preoperative period were in complete remission. One of 20 patients who was started to use oral antidiabetic drugs during preoperative period was in partial remission.

At the 12th month of postoperative follow-up 29 of 33 patients who were using insulin were in complete remission. One of 33 patients who were using insulin were in partial remission. Three of 33 patients, who were using insulin were not in remission. 54 of the 55 patients who were using oral antidiabetic drugs were in complete remission and 1 of 55 patients was in partial remission.

Totally, complete remission was observed in 90.9 %, partial remission was observed in 3.4 % and persistent diabetes in 5.6 % of the patients at the 6th month. At 12th month complete remission was observed in 94.3 %, partial remission was observed in 2.2 % and persistent diabetes in 3.4 % of the patients (Table 3).

**Table 3:** Type 2 diabetes complete remission

	Postoperative 6th month (%)	Postoperative 12th month (%)
Using insulin	78.78	87.87
Using oral antidiabetic drugs	96.36	98.18

Diabetes age of the 3 persistent diabetics was 80.33 ± 8.02 months, diabetes age of the 2 diabetics having partial remission was 57 ± 4.24 months and diabetes age of the patients achieving complete remission was 22.22 ± 11.71 months.

In our univariate logistic regression analysis, patient age, postoperative (PO) 6th month EWL, PO 12th month EWL, PO 6th month BMI and PO 12th month BMI were emerged as the decisive parameters (Table 4). In multivariate logistic regression analysis (forward type) patient age and PO 6th month EWL were emerged as the decisive parameters (Table 5).

**Table 4:** Factors affecting diabetes remission (univariate)

	p	OR	95 % CI	
			Min	Max
Patient age	*0.003*	1.320	1.097	1.589
BMI	0.068	1.093	0.993	1.203
DM AGE	0.055	1.271	0.994	1.623
Usage of insulin	0.079	7.448	0.795	69.774
Usage of OAD	0.079	0.134	0.014	1.258
PO 6th month EWL	*0.007*	0.756	0.617	0.926
PO 12th month EWL	*0.003*	0.649	0.490	0.859
Po 6th month BMI	*0.005*	1.446	1.119	1.870
PO 12th month BMI	*0.004*	2.073	1.260	3.408

Univariate logictic regression analysis

Italic values are statistically meaningful (p<0.05)

**Table 5:** Factors affecting diabetes remission (multivariate)

	p	OR	95 % CI	
Patient age	0.019	1.447	1.062	1.971
PO 6th month EWL	0.048	0.608	0.371	0.996

*Model:* Patient Age, PO 6th month EWL, PO 12th month EWL, PO 6th month BMI, PO 12th month BMI

Multivariate logictic regression analysis (Forward)

In one patient postoperative leakage was observed and it was treated with percutaneous drainage inserted by a radiologist, self-expandable stents were not required. In another patient postoperative bleeding occurred and was treated with laparoscopic hematoma drainage. No perioperative or postoperative mortality was seen.

## Discussion

Obesity is one of the major health problems in this era and endocrinological problems as type 2 diabetes may be one of its consequents (Standards of Medical Care in Diabetes–2012). Adequate medical treatment of type 2 diabetes is difficult to achieve, whereas bariatric surgery is proven to be a sufficient treatment method of not only weight loss but also endocrinological diseases in the morbid obese patients (Buchwald et al. 2009). In this study, our results supported this knowledge. We found, that complete remission in type 2 diabetes was observed after sleeve gastrectomy in a follow-up of 6 months as 90.9 % and 1 year as 95.4 %; previously 37.5 % of these patients were insulin dependent and 62.5 % were using oral antidiabetic drugs.

Previously reported diabetes remission rates change from 40 to 90 %, which vary related to the operation type, as diabetes remission is recently reported as 60–90 % in RYGBP, 86 % in bilio-pancreatic diversion (BPD); the long term results are 60 % diabetes remission in RYGBP and SLG and 50–70 % in insulin dependent diabetes (Boza et al. 2011; Cordera and Adami 2016; Miras et al. 2014). Variations in diabetes remission rates may be explained by the usage of different remission criteria of ADA, which were upgraded in different years. We used the 2012 diabetes remission criteria (Standards of Medical Care in Diabetes–2012). The results in our study were quite better compared to the previous studies concerning diabetes remission (Miras et al. 2014; Rubino et al. 2010; Bayham et al. 2012). This may be parallel to the success of our hospital’s bariatric surgery team, as all patients in our study group were evaluated by this team, which is interdisciplinary consisting of endocrinologists, general surgeons, anesthesiologists, psychiatrists, pulmonologists, nutritionists and cardiologists. Morbid obese patients underwent surgery only after the approval of the bariatric surgery team. The patients were already treated by nutritionists and endocrinologists for at least 1 year before being eligible for further evaluation of the bariatric surgery team. After the surgical procedure patients were followed up by nutritionists and surgeons closely. A strict diet was ordered postoperatively. Besides, our patients established an active social network between each other, their nutritionists and their surgeons. We believe, that sleeve gastrectomy with 36 F bougie, total antral and fundus resection together with postoperative close follow-up, strict diet and motivating active social network helped to achieve our results.

Bariatric surgery includes a lot of surgical techniques, but laparoscopic interventions are preferred by surgeons, because of reduced complications, shorter postoperative recovery period and hospital stay. For similar reasons, among various surgical procedures LSG is a simple and quite effective surgical technique in the treatment of morbid obesity (Zhang et al. 2015). Recently laparoscopic sleeve gastrectomy has been reported to be effective in the complete remission of type 2 diabetes (Lemanu et al. 2015). But larger sample sizes are necessary to make more accurate conclusions. In this study we followed up 88 patients having type 2 diabetes and reported their results after postoperative one-year follow-up.

During the preoperative assessment, diagnosis of type 2 diabetes and also the treatment modality should be carefully questioned. Even if the patient does not have a history of type 2 diabetes preoperative laboratory tests should include HbA1c. This laboratory test may help to detect an undiagnosed of type 2 diabetes (Razi et al. 2015). In our clinical preoperative assessment protocol, we routinely check for elevated HbA1c, which also indicates whether diabetes is treated well and blood glucose is in normal ranges. In our patients preoperatively HbA1c was 7.33 ± 1.24 %, HbA1c at the postoperative 6th month check was 5.5 ± 0.7 % and HbA1c at the postoperative 12th month check was 5.2 ± 0.33 %. One of the limitations in this study was, that we did not measure C-peptide preoperatively and postoperatively because of financial problems in our hospital.

Medical treatment status before surgery is another important factor to consider (Peterli et al. 2009; Wu et al. 2015). Preoperative insulin dependent 33 diabetic patients were in complete remission up to 87.87 % in our study and 55 patients receiving oral antidiabetic treatment were in complete remission up to 98.18 %. Besides, we believe that the period of using insulin or oral antidiabetic drugs and the age of diabetes is also important on the treatment success of type 2 diabetes. Diabetes age of the 3 persistent diabetics was 80.33 ± 8.02 months, diabetes age of the 2 diabetics having partial remission was 57 ± 4.24 months and diabetes age of the patients achieving complete remission was 22.22 ± 11.71 months. It can be concluded, that the earlier type 2 diabetes is diagnosed, the better diabetes remission can be achieved. In our country laparoscopic sleeve gastrectomy is paid by health insurances only in patients with a BMI over 40 kg/m^2^. In our opinion, obesity surgery should be done at lower BMI as <35 kg/m^2^ in type 2 diabetics to increase diabetes remission success (Kular et al. 2015).

It is still not known, how glycemic regulation after bariatric surgery works exactly. Weight loss is estimated to be one of the strong factors, glycemic control success increases parallel to EWL (Lee et al. 2013). Early regulation of the FGL is unclear. A lot of influencing factors have to be investigated. Our study data supported this knowledge, as preoperative BMI of the 88 patients was 48.65 ± 7.71 kg/m^2^, BMI at the 6th month was 30 ± 3.28 kg/m^2^ and BMI at the 12th month was 26 ± 2.53 kg/m^2^ with a 6th month EWL of 68 ± 5.87 % and 12th month EWL of 81 ± 4.98 %.

In previous studies shorter diabetes age was associated with higher diabetes remission rate (Yu et al. 2015). In our study although diabetes age of the 3 persistent diabetics was 80.33 ± 8.02 months, diabetes age of the 2 diabetics having partial remission was 57 ± 4.24 months and diabetes age of the patients achieving complete remission was 22.22 ± 11.71 months, univariate logistic regression analysis indicated that patient age, PO 6th month-EWL, PO 12th month EWL, PO 6th month BMI and PO 12th month BMI were emerged as the decisive parameters of DM remission and diabetes age was not statistically significant. In multivariate logistic regression analysis (forward type) patient age and PO 6th month-EWL were emerged as the decisive parameters of DM remission. In previous systemic review and meta-analysis EWL was not correlated with DM remission rate and in our study patient age and PO 6th month-EWL were emerged as the decisive parameters of diabetes remission (Cho et al. 2015). These differences might be explained with a little higher EWL, all patients were BMI > 40 kg/m^2^ with preoperative BMI 48.65 ± 7.71 kg/m^2^, a younger patient population with age 36 ± 9.3, a strict diet and motivating active social network in our study compared to previous studies. Finally, only 5 patients were not in complete remission whereas 83 patients were in complete remission. This might have changed the regression analysis results.

 This study has promising results in the surgical treatment of Type 2 DM with a 94.3 % complete remission rate. However, there are some limitations as short fallow up time (16.09 ± 3.32 months), small patient population (88 patients) and inclusion of new diagnosed patients (20 patients) in this study. Collection of data is still continuing in our clinic to observe long term results.^1^

## Conclusion

Sleeve gastrectomy causes minimal physiological changes in the gastrointestinal tract and provides sufficient weight loss at the same time. Additional benefits are glycemic control and remission in type 2 diabetes. It seems to be a good solution to treat type 2 diabetics surgically.

## Abbreviations

LGS: laparoscopic Sleeve Gastrectomy
RYGBP: Roux-en-Y Gastric By-pass
ADA: American Diabetes Association
BMI: body mass index (kg/m^2^)
FGL: fasting glucose level (g/dL)
HbA1C: glycosylated hemoglobin (%)
SD: standard deviation
%WL: percentage of body weight loss
%EWL: percentage of excess body weight loss
PO: postoperative

## Footnotes

1 Treatment of morbid obesity and comorbidities is quite challenging, if only treated medically. However, surgical interventions as laparoscopic sleeve gastrectomy make it easier to treat morbid obesity and endocrinological diseases related to that.
